# Evaluation and limitations of different approaches among COVID-19 fatal cases using whole-exome sequencing data

**DOI:** 10.1186/s12864-022-09084-5

**Published:** 2023-01-10

**Authors:** Natalia Forgacova, Zuzana Holesova, Rastislav Hekel, Tatiana Sedlackova, Zuzana Pos, Lucia Krivosikova, Pavol Janega, Kristina Mikus Kuracinova, Pavel Babal, Peter Radvak, Jan Radvanszky, Juraj Gazdarica, Jaroslav Budis, Tomas Szemes

**Affiliations:** 1 Comenius University Science Park, Bratislava, 841 04 Slovakia; 2 Faculty of Natural Sciences, Comenius University, Bratislava, 841 04 Slovakia; 3 Institute of Clinical and Translational Research, Biomedical Research Centre, Slovak Academy of Sciences, Bratislava, 845 05 Slovakia; 4 Geneton Ltd, Bratislava, 841 04 Slovakia; 5 Slovak Centre of Scientific and Technical Information, Bratislava, 811 04 Slovakia; 6 Department of Pathology, Faculty of Medicine, Comenius University, Bratislava, 813 72 Slovakia

**Keywords:** SARS-CoV-2, COVID-19, Whole-exome sequencing, Genetic association, Polymorphisms, Gnomad, Non-invasive prenatal testing

## Abstract

**Background:**

COVID-19 caused by the SARS-CoV-2 infection may result in various disease symptoms and severity, ranging from asymptomatic, through mildly symptomatic, up to very severe and even fatal cases. Although environmental, clinical, and social factors play important roles in both susceptibility to the SARS-CoV-2 infection and progress of COVID-19 disease, it is becoming evident that both pathogen and host genetic factors are important too. In this study, we report findings from whole-exome sequencing (WES) of 27 individuals who died due to COVID-19, especially focusing on frequencies of DNA variants in genes previously associated with the SARS-CoV-2 infection and the severity of COVID-19.

**Results:**

We selected the risk DNA variants/alleles or target genes using four different approaches: 1) aggregated GWAS results from the GWAS Catalog; 2) selected publications from PubMed; 3) the aggregated results of the Host Genetics Initiative database; and 4) a commercial DNA variant annotation/interpretation tool providing its own knowledgebase. We divided these variants/genes into those reported to influence the susceptibility to the SARS-CoV-2 infection and those influencing the severity of COVID-19. Based on the above, we compared the frequencies of alleles found in the fatal COVID-19 cases to the frequencies identified in two population control datasets (non-Finnish European population from the gnomAD database and genomic frequencies specific for the Slovak population from our own database). When compared to both control population datasets, our analyses indicated a trend of higher frequencies of severe COVID-19 associated risk alleles among fatal COVID-19 cases. This trend reached statistical significance specifically when using the HGI-derived variant list. We also analysed other approaches to WES data evaluation, demonstrating its utility as well as limitations.

**Conclusions:**

Although our results proved the likely involvement of host genetic factors pointed out by previous studies looking into severity of COVID-19 disease, careful considerations of the molecular-testing strategies and the evaluated genomic positions may have a strong impact on the utility of genomic testing.

**Supplementary Information:**

The online version contains supplementary material available at 10.1186/s12864-022-09084-5.

## Background

The coronavirus disease (COVID-19), caused by the severe acute respiratory syndrome coronavirus 2 (SARS-CoV-2), is a complex, highly infectious disease involving the respiratory, immune, cardiovascular, gastrointestinal, and neurological systems [1–4]. The first case was registered in Wuhan, Hubei Province of China in December 2019, and the disease has rapidly evolved into a global pandemic [5]. At the time of writing (February 2022), there have been more than 410 million confirmed cases and 5.8 million deaths worldwide (in Slovakia, more than 1.8 million people have been infected so far, with total death toll exceeding 18 000) (https://origin-coronavirus.jhu.edu/map.html).

The mortality rate of COVID-19 (ranges between 1–7%) is lower than that of the other two types of coronaviruses, the severe acute respiratory syndrome (SARS-CoV) and the middle east respiratory syndrome (MERS-CoV). However, the rate of human-to-human transmission is higher, as the virus can primarily be transmitted through respiratory droplets and close contact [4, 6–10]. COVID-19 presents a broad spectrum of varied clinical manifestations, from asymptomatic or mild symptoms to serious health outcomes leading to death [11, 12]. Even though the symptoms are highly heterogeneous, the most commonly observed ones in the large majority of infected persons include fever, cough, severe headache, muscle pain, fatigue, shortness of breath, chest tightness, and loss of taste or smell [13–18]. Besides, several minor symptoms such as gastrointestinal complications, including nausea, vomiting, and diarrhea, have also been reported [19]. In severe cases, breathing difficulties develop, namely dyspnea, with acute respiratory distress syndrome (ARDS) being the most serious complication [20].

SARS-CoV-2 infection exhibits varied infectivity and mortality rates in different worldwide populations [21, 22]. An obvious possible explanation for these findings is that a mixture of genetic and nongenetic factors interplays between the virus and host genetic background, which determines the severity of COVID-19 outcome. Older age (> 60 years old), male gender, blood type, smoking, hypertension, diabetes mellitus, obesity, cardiovascular, respiratory, and kidney disease or cancer have been identified as risk factors associated with a higher risk of death caused by COVID-19 [12, 23–29]. In addition, the host’s susceptibility or response to the infection can be influenced by the host's genetic variations affecting the structure or function of essential proteins playing an active role in the entry and spread of SARS-CoV-2. This assumption is supported by a study of twins reporting 50% heritability of COVID-19 risk [30].

In this respect, the COVID-19 Host Genetics Initiative (HGI), 2020 (available at https://www.covid19hg.org/) is currently leading a worldwide public effort to analyse COVID-19 information in millions of individuals in relation with genotype data. It aims to identify genetic variants associated with SARS-CoV-2 infection as well as COVID-19 rate of hospitalization and disease severity. A recent study by Baggen et al. summarizes the wealth of information on proviral SARS-CoV-2 host factors that have been produced by genome-wide functional genetic screens and interactome analyses with further discussion about their roles in cellular processes [31]. Moreover, worldwide genome-wide association studies (GWAS) have identified many risk genes and loci that could be functionally implicated in COVID-19 disease [32–36]. Recent whole-exome sequencing (WES)-based studies in several countries also aimed to identify the genetic factors of COVID-19 susceptibility. However, it is to be noted that these studies are focused only on investigating genetic variants in small groups of risk genes, mainly related to the initial stages of infection [37–42].

In this study, we summarized available resources of known risk variants and genes associated with COVID-19 to explain patients’s genetic predispositions for a severe course or higher mortality of COVID-19. We collected and analysed genomic variants that either: a.) showed evidence of association with COVID-19 in GWAS studies, b.) were located in genes previously reported to be a risk in selected studies by literature search, c.) showed strong association in meta-analyses conducted by the COVID-19 HGI Browser, and d.) were located in genes associated with COVID-19 by QIAGEN Clinical Insight (QCI™) Analyze software. We present a comprehensive comparative assessment of variants identified in these four groups compared to our cohort of genotyped patients. In addition, allele frequencies of identified variants were compared with two types of control groups, worldwide public data of the non-Finnish European population from the gnomAD database (NFE) and our genetic data from Non-Invasive Prenatal Testing (NIPT) in the Slovak population. Despite the growing knowledge about genetic factors associated with the risk of COVID-19, there is a lack of studies that elucidate the multitude of other factors that influence this disease. In this study, we provide different approaches to analysing genetic variants in the WES data from Slovak patients who have died of COVID-19.

## Results

### Analyses of variants associated with COVID-19 in GWAS Catalog

The first goal of our analysis was to investigate the variants associated with COVID-19 in GWAS Catalog (https://www.ebi.ac.uk/gwas/). Overall, 413 risk variants in a total of 403 genes were identified in the GWAS dataset after entering the keyword “COVID-19” into the GWAS Catalog Browser. After merging all identified variants from GWAS (413 risk variants) with our WES data of the deceased patients, we identified only 3 common variants – the rs11147040 missense variant, the synonymous rs72472161 variant, and the rs8176719 coding sequence variant. Other identified variants belonged to the intron, non-coding, and UTR variants group.

### Analyses of COVID-19 risk variants through literature search

After conducting the literature search, we chose 3 types of studies: I. a study by Baggen et al. [31] included cellular host factors for SARS-CoV-2 infection, II. studies by COVID-19 HGI [43] and Pairo-Castineira et al. [35], which reported genome-wide loci associated with severe manifestations of COVID-19, and III. Ackermann et al. [44] which analysed respiratory failure-associated gene sets (Table S1).

**Fig. 1:**
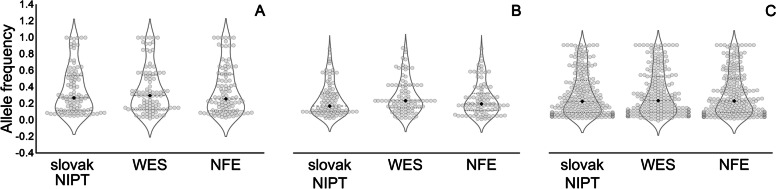
Violin-swarm plots show allele frequencies for the Slovak NIPT and WES data and NFE in risk variants identified in 3 types of publications: I. Baggen et al. [31] (103 risk variants). Statistic: median_sN = 0.280, sd_sN = 0.285; median_W = 0.296; sd_W = 0.284; median_*N* = 0.265, sd_*N* = 0.291; Mann–Whitney U test (WES vs. Slovak NIPT): *p*-value = 0.3539; Mann–Whitney U test (WES vs. NFE): *p*-value = 0.4146. II. COVID-19 Host Genetics Initiative [43] and Pairo-Castineira et al. [35] (96 risk variants). Statistic: median_sN = 0.21, sd_sN = 0.215; median_W = 0.278; sd_W = 0.223; median_N = 0.238, sd_*N* = 0.212; Mann–Whitney U test (WES vs. Slovak NIPT): *p*-value = 0.0518; Mann–Whitney U test (WES vs. NFE): *p*-value = 0.0816. III. Ackermann et al. [44] (245 risk variants). Statistic: median_sN = 0.268, sd_sN = 0.281; median_W = 0.278; sd_W = 0.287; median_*N* = 0.274, sd_*N* = 0.283; Mann–Whitney U test (WES vs. Slovak NIPT): *p*-value = 0.4048; Mann–Whitney U test (WES vs. NFE): *p*-value = 0.3854. Median is labeled with ♦; sd—standard deviation, sN—Slovak NIPT, W—WES—whole-exome sequencing data from the deceased patients, N—Non-Finnish European population from the gnomAD database

When we merged all identified variants with the WES data of the deceased Slovak patients, we identified 208 (I.), 154 (II.), and 437 (III.) common risk variants, respectively. Next, we compared this data with two control groups—the NIPT data from the Slovak population, where we identified 103 (I.), 96 (II.), and 245 (III.) common risk variants and the NFE data (Table S2, Figure S1). Using the NFE data, we performed two types of analyses, first with risk variants found in the overlap publications data/WES/NIPT and the second with risk variants from the overlap of the publications data/WES. The violin-swarm plots in Fig. 1 represent the graphical comparison of the allele frequencies of risk variants from the overlap of the publications data/WES/NIPT/NFE (103, 96, and 245 common risk variants). The comparison of allele frequencies of risk variants from the overlap of the publication data/WES/NFE (208, 155, and 437 risk variants) is shown in Figure S2. Using the Mann–Whitney U test, we did not identify any significant difference between the groups in both types of comparisons.

Subsequently, allele counts of risk variants identified in our sample sets from the WES data were compared to allele counts of these variants in the Slovak NIPT data and the NFE data using the Fisher's exact test. By comparing risk variant allele counts in individual groups of publications (I., II., III.), we have identified 9, 12, and 10 risk variants with *p*-value under 0.05 in the comparison between the NIPT and the WES data and 4, 21, and 15 risk variants in the comparison between the WES and NFE data, respectively (Table S2). After implementing the Bonferroni correction, the rs35048651 risk variant was identified with a significantly different representation (*p*-value = 0.000005) in the I. group. Also, we identified the rs2498800 risk variant (*p*-value = 0.000035) in the III. group after comparison between the NIPT and WES data. Further still, the rs7853989, rs8176741, rs8176743, rs8176746, rs8176747, rs8176749, and rs8176751 risk variants were found in the II. group based on comparison between the WES and NFE data in the overlap of the publications data/WES/NIPT/NFE (Table 2). Using the Fisher's exact test, we also compared allele counts of risk variants from the overlap of the publications data/WES/NFE. Here, we found 43, 30, and 92 risk variants, respectively with a *p*-value under 0.05. After the Bonferroni correction, we identified the rs78472109, rs4024453, rs594178, rs146238849, rs62478356, rs10454320, rs56850341, rs150756699 and rs377124451 risk variants in the I. group. The same process led to identification of rs8176751, rs8176749, rs8176747, rs8176746, rs8176743, rs8176741 and rs7853989 risk variants in the II. group as well as rs373030497, rs9521779, rs1226613997, rs540909253, rs9521780, rs9515217, rs201005516 and rs201495169 risk variants in the III. group all characterised by a significantly different representation (Table S2).

### Analyses of COVID-19 risk variants using the COVID-19 Host Genetics Initiative

In this part, we analysed the COVID-19 risk variants identified in meta-analyses conducted by the COVID-19 HGI Browser. We focused only on missense variants from all four genome-wide meta-analysis results available online (https://app.covid19hg.org/). The total number of COVID-19 missense risk variants found in each HGI group is summarized in Table 1.

**Table 1:** The total number of risk variants that were common across the individual comparative analysis (between NIPT, WES data and variants found in publications and individual HGI groups). *Comparison results from NIPT/WES/NFE/pub or HGI data/QCI analysis published in Results; ** Comparison results from NFE/WES/pub or HGI data/QCI analysis published in supplementary files; *** % of variants from comparison total number of NIPT/WES/Covid-19 HGI risk variants to the total number of risk variants in HGI group; **** % of variants from comparison number of WES/NFE/QCI/pub or HGI data risk variants to the total number of risk variants in comparison: WES/NFE/pub or HGI data; ***** % of variants from comparison number of NIPT/WES/NFE/QCI/pub or HGI data risk variants to the total number of risk variants in comparison: NIPT/WES/pub or HGI data

	**Source of data**	**The total number of all variants (publications)/The total number of missense risk variants (COVID-19 HGI Group)**	The number of risk variants in comparison: WES/NFE **	The number of risk variants in comparison: NIPT/WES/pub or HGI data * (in parentheses ***)	**QCI analysis—infection group (376 risk variants)**		**QCI analysis—critical group (1738 risk variants)**	
					Risk variants in comparison: WES/NFE/pub or HGI data ** (in parentheses ****)	Risk variants in comparison: NIPT/WES/NFE/pub or HGI data * (in parentheses *****)	Risk variants in comparison: WES/NFE/pub or HGI data ** (in parentheses ****)	Risk variants in comparison: NIPT/WES/NFE/pub or HGI data * (in parentheses *****)
**Publications (pub)**	**I: infection**	1,745,372	208	103	0 (0%)	0 (0%)	0 (0%)	0 (0%)
	**II: severity**	277,165	154	96	0 (0%)	0 (0%)	14 (9.1%)	7 (7.3%)
	**III: respiratory failure**	3,076,573	437	245	0 (0%)	0 (0%)	43 (9.8%)	22 (9%)
**COVID-19 HGI group (HGI)**	**A2: critically ill Covid-19 + vs. population**	62	51	45 (72.6%)	3 (5.9%)	2 (4.4%)	1 (2%)	1 (2.2%)
	**B1: hospitalized Covid-19 + vs. non-hospitalized Covid-19 + **	16	13	13 (81.3%)	0 (0%)	0 (0%)	0 (0%)	0 (0%)
	**B2: hospitalized Covid-19 + vs. population**	72	58	51 (70.8%)	3 (5.2%)	2 (3.9%)	1 (1.7%)	1 (2%)
	**C2: reported SARS-CoV-2 infected vs. population**	83	63	52 (62.7%)	0 (0%)	0 (0%)	13 (20.6%)	9 (17.3%)

**Fig. 2:**
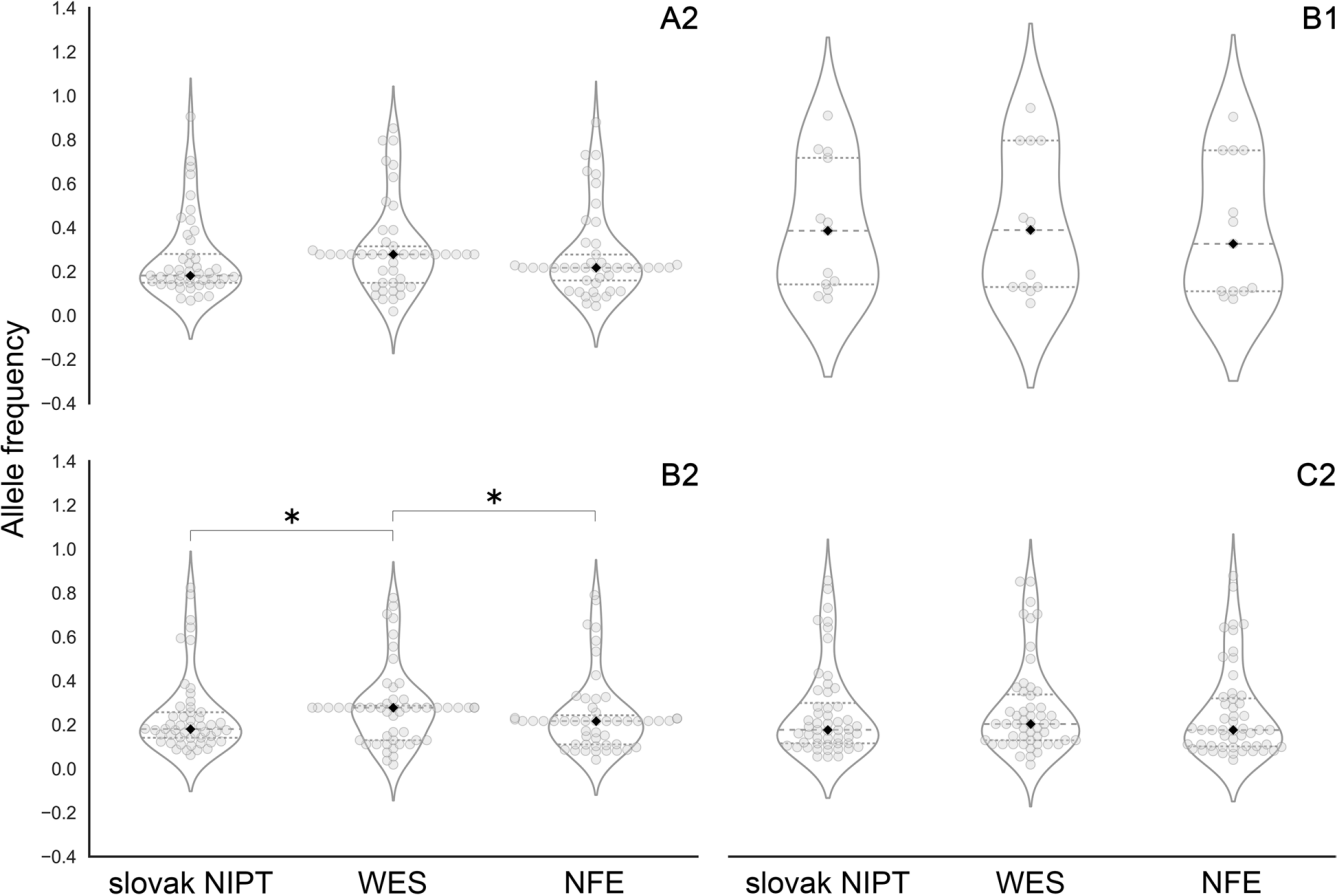
Violin-swarm plots show allele frequencies for the Slovak NIPT and WES data and NFE data. Statistical significance is labeled with *, median with ♦. A2: Critically ill vs. population controls (45 risk variants). Statistics: median_W = 0.278; sd_W = 205; median_sN = 0.182, sd_sN = 0.186; median_*N* = 0.217, sd_*N* = 0.198; Mann–Whitney U test (WES vs. Slovak NIPT): *p*-value = 0.06510; Mann–Whitney U test (WES vs. NFE): *p*-value = 0.05363. B1: Hospitalized COVID-19 vs. non-hospitalized COVID-19 (13 risk variants). Statistics: median_W = 0.389; sd_W = 0.321; median_sN = 0.386, sd_sN = 0.297; median_*N* = 0.326, sd_*N* = 0.312; Mann–Whitney U test: WES vs. Slovak NIPT: *p*-value = 0.41863; WES vs. NFE: *p*-value = 0.22057. B2: Hospitalized COVID-19 vs population controls (51 risk variants). Statistics: median_W = 0.278; sd_W = 0.179; median_sN = 0.181, sd_sN = 0.181; median_*N* = 0.217, sd_*N* = 0.176; Mann–Whitney U test: WES vs. slovak NIPT: *p*-value = 0.04538; WES vs. NFE: *p*-value = 0.01872. C2: Reported SARS-CoV-2 infections vs. population controls (52 risk variants). Statistics: median_W = 0.204; sd_W = 0.21; median_sN = 0.177, sd_sN = 0.207; median_*N* = 0.176, sd_*N* = 0.209; Mann–Whitney U test: WES vs. slovak NIPT: *p*-value = 0.26942; WES vs. NFE: *p*-value = 0.12153; sd—standard deviation, sN—Slovak NIPT, W—WES—whole-exome sequencing data from deceased patients, NFE—Non-Finnish European population from the gnomAD database

After merging all missense risk variants identified in individual HGI groups with the WES data of the deceased patients, we determined 51 common risk variants in the A2 group, 13 risk variants in the B1 group, 58 risk variants in the B2 group, and 63 risk variants in the C2 group (Table S2). This data was compared with two controls – the first represented the Slovak NIPT data, where we identified 45 common risk variants in the A2 group, 13 in the B1 group, 51 in the B2 group and 52 in the C2 group (Table S2, Figure S1). The second was the NFE data, where we performed two different analyses, first with the risk variants that were found in the overlap HGI groups data/WES/NIPT, and the second with risk variants from the overlap of HGI groups data/WES. The violin-swarm plots displaying graphical comparison of the allele frequencies of risk variants from the overlap HGI groups data/WES/NIPT/NFE are shown in Fig. 2. Likewise, the graphical comparison of the allele frequencies of risk variants from the overlap HGI groups data/WES/NFE is shown in Figure S3.

Using the Mann–Whitney U test, we observed a significant difference in the comparison between the WES data and the NIPT data (*p*-value = 0.02609) as well as between the WES data and the NFE data (*p*-value = 0.01872), both in the B2 group in the HGI groups data/WES/NIPT/NFE overlap (Fig. 2). Another significant difference was identified between the WES data and NFE data in the B2 group in the HGI groups data/WES/NFE overlap (*p*-value = 0.03132, Figure S3). The differences identified in the other comparisons were negligible.

Using the Fisher's exact test, we compared the allele count of risk variants identified in our sample set from the WES data to allele counts of these variants in the Slovak NIPT data as well as the NFE data. We identified 3 variants in the A2 group, 4 variants in the B2, and 2 risk variants in the C2 group when comparing the WES and NIPT data. Likewise, we found 2 risk variants in the A2 group, 2 risk variants in the B2 group, and 7 risk variants in the C2 group in the comparison between the WES and NFE data with *p*-value under 0.05 in the overlap HGI groups data/WES/NIPT/NFE (Table S2). After the Bonferroni correction, the rs3130984 variant was identified with a significantly different representation (*p*-value = 0.000056) in the comparison between the WES and NIPT data in the A2 group. Equally, 4 risk variants, rs8176747, rs8176746, rs8176743 and rs7853989 (*p*-value = 0.00025) were identified in the comparison between the WES and NFE data in the C2 group (Table 2). We did not observe any significant differences in the B1 and B2 groups. After applying the Fisher's exact test to compare the allele count of risk variants in the overlap of HGI groups data/WES/NFE, we found 2 risk variants in the A2 group, 2 risk variants in the B2 group, and 8 risk variants in the C2 group with *p*-value under 0.05. After the Bonferroni correction, the results correspond with the figures from the comparison between WES and NFE in the overlap of HGI groups data/WES/NIPT/NFE.

**Table 2:** Risk variants with a significant difference (after the Bonferroni correction) in allele distribution of WES data from patients who died of Covid-19 and the control data of the Slovak NIPT (W/sN) and NFE (W/N). sig—significance: *—*p*-value < 0.05, **—significant after the Bonferroni correction

					**Fisher's exact test *p*-values in WES/Slovak NIPT (W/sN) and the WES/NFE comparisons (W/N)**																			
					**Publications**												**COVID-19 HGI browser**							
					**Infection**				**Disease severity**				**Respiratory failure**				**A2: critically ill vs. pop**				**C2: reported infect. vs. pop**			
	**Variant ID**	**Risk variant**	**Gene**	**Consequence**	**sig**	**W/sN**	**sig**	**W/N**	**sig**	**W/sN**	**sig**	**W/N**	**sig**	**W/sN**	**sig**	**W/N**	**sig**	**W/sN**	**sig**	**W/N**	**sig**	**W/sN**	**sig**	**W/N**
**1**	6–31,117,187-T-C	rs3130984	PSORS1C1	Missense variant													**	0.000056		0.356741				
**2**	9–133,255,801-C-T	rs8176749	ABO	Non coding transcript exon variant						0.106383	**	0.000253												
**3**	9–133,255,928-C-G	rs8176747	ABO	Missense variant						0.575117	**	0.000253										0.575117	**	0.000253
**4**	9–133,255,935-G-T	rs8176746	ABO	Missense variant						0.258419	**	0.000252										0.258419	**	0.000252
**5**	9–133,256,028-C-T	rs8176743	ABO	Missense variant					*	0.027003	**	0.000253									*	0.027003	**	0.000253
**6**	9–133,256,074-G-A	rs8176741	ABO	Non coding transcript exon variant						0.237448	**	0.000262												
**7**	9–133,256,205-G-C	rs7853989	ABO	Missense variant						0.363999	**	0.000252										0.363999	**	0.000252
**8**	9–133,255,635-C-T	rs8176751	ABO	Non coding transcript exon variant					*	0.026342	**	0.000246												
**9**	14–104,772,267-C-T	rs2498800	AKT1	Intron variant									**	0.000035		0.376467								
**10**	17–1,728,046-TGAG-T	rs35048651	WDR81	5 Prime UTR variant	**	0.000005		0.136225																

### Analyses of variants by QIAGEN Clinical Insight (QCI™) Analyze software

After analysing the variant call format files (VCF) for each patient and then applying a biological filter, we selected 4 from the 8 offered COVID-19 filters. Variants are divided into two categories to follow the concept applied in other analyses: the QCI infection group—containing 376 risk variants with the "Mild COVID-19" biological filter and the QCI critical group containing 1738 variants with "Critical COVID-19", "Severe COVID-19" and "COVID-19 related immunodeficiency 74" biological filters. Other filters were excluded from further analyses as it was not possible to clearly determine their classification into infected or critical groups. Subsequently, a number of risk variants in the WES/NFE comparison or in the NIPT/WES/pub comparison or HGI data were contrasted to the QCI analysis of the infection and critical groups. The results of individual comparisons of the number of variants are shown in Table 1.

## Discussion

In the present article, we attempted to elaborate on the spectrum of risk variants and genes identified in different ways and their possible relationship to severity and/or mortality of COVID-19. We investigated the frequencies of these variants and evaluated their possible role using a cohort of 27 Slovak patients who have died of COVID-19. In our previous studies, we described the re-use of the data from NIPT for genome-scale population-specific frequency determination of small DNA variants [45], copy number variants (CNVs) [46], and variants associated with colorectal cancer and Lynch syndrome [47]. Therefore, we assumed that the NIPT data can be utilized as a control group in this population study of COVID-19. As a second control group, we selected the NFE genetic data which contains a total of 125,748 exomes and 71,702 genomes. To our knowledge, the present study is the first population analysis of COVID-19 variants worldwide and also in the Slovak population that provides different approaches to the analysis of genetic variants in the WES data of patients who have died of COVID-19.

Over the past two years, GWAS have offered the opportunity to reveal factors of genetic susceptibility factors to COVID-19 disease and provided insights into the biological basis of SARS-CoV-2 etiology. To date, a large number of risk genetic variants and genes have been identified by the GWAS approach, which has been intimately connected to the COVID-19 susceptibility and severity [32–35, 48]. As we only identified 3 common variants between the GWAS catalog and our WES data, we have found that risk variants from the GWAS catalog are not useful for analyzing and comparing our WES-derived data.

In 2021, a large consortium conducted some highly anticipated studies published last year. The COVID‐19 HGI presented results from three genome‐wide association meta‐analyses of up to 49,562 COVID‐19 patients from 46 studies spanning across 19 countries [49]. They reported 13 genome‐wide significant loci. The 3p21.31 region seemed to be associated with infection susceptibility, which was also confirmed in a study by Ellinghaus et al. This study also confirmed a potential involvement of the ABO blood-group system [32]. Similar results were also found in a study conducted by 23andMe company using their own biobank. After the Bonferroni correction in the analysis of COVID-19 missense risk variants conducted by the COVID-19 HGI Browser, we identified 5 variants with a significantly different representation; the rs3130984 missense variant located in the *CDSN* (Corneodesmosin) gene and four variants (rs8176747, rs8176746, rs8176743, and rs7853989) all located in the *ABO* gene. Two missense variants, rs8176747 and rs8176746, were found by a comparison between the WES and NFE data in the C2 HGI group and the II. group in the analysis using literature search. Recently, a GWAS found a COVID-19-association signal at locus 9q34.2 coincident with the *ABO*-blood group (rs8176747, rs41302905, rs8176719) in Italian and Spanish severe COVID-19 patients with respiratory failure [32, 50]. Another study published a genetic hypothesis on the role of the renin-angiotensin system (RAS) genes—*ACE1* (Angiotensin-converting enzyme 1), *ACE2* (Angiotensin-converting enzyme 2), and *ABO*-locus (rs495828, rs8176746) in COVID-19 prognosis, suspecting inherited genetic predispositions to be predictive of COVID-19 severity [51].

Since 2017, the QIAGEN Clinical Insight (QCI™) Analyze software has been available as an integrated clinical decision support solution for the annotation, interpretation and reporting of NGS data. QCI seeks to extract clinical significance and actionability from sequencing data [52]. After a literature search of studies using QIAGEN Clinical Insight (QCI™) Analyze software, we found only a few studies focusing mainly on cancer research [52–54]. As anticipated, a comparison of the QCI results showed an overlap in the number of risk variants found in the QCI critical group with the variants in the groups II. and III. that publications associated with the severity and mortality of COVID-19. However, we did not identify any overlap with the group I.; the publication included cellular host factors for SARS-CoV-2 infection. In the B1 HGI group, no overlap was found in the number of variants with the QCI infection and the critical group, which may be due to the relatively small number of identified missense variants (only 13 risk variants). In the C2 HGI group, we expected to overlap with both groups, but we only identified with the QCI critical group. Although usage of the QIAGEN Clinical Insight (QCI ™) Analyze software requires further optimization, this software may be a promising diagnostic, prognostic, or predictive tool in the future research of COVID-19.

Our study has several key shortcomings. First, although we used different approaches to analyse the WES data of patients who died of COVID-19, we were able to identify only 10 risk variants with significant difference (after the Bonferroni correction) in allele distribution of the WES data and data of Slovak NIPT and NFE. Next, the sample size of the deceased patients was relatively small. Moreover, data from NIPT are strongly biased towards the healthy population of females. It should also be noted that the total number of analyzed risk variants identified by COVID-19 HGI was also relatively small as we focused only on missense variants. We targeted missense variants to identify as many risk variants as possible since most of the identified variants from HGI belonged to the non-coding category. In GWAS analysis, this proved to be useless for analysis of our WES data.

## Conclusions

As the COVID-19 pandemic led to a global crisis with a serious global impact, it is crucial to determine how host genetic factors are linked to the clinical outcomes. Therefore, many studies have focused on discovering the influence of host genetic factors on the SARS-CoV-2 infection risk or COVID-19 severity. The main aim of our study was to provide an informative insight into the utility of COVID-19 data from publicly available databases with practical application to WES data from COVID-19 patients. WES is routinely used and is gradually being optimized for the detection of rare and common genetic variants in humans. Genetic stratification of patients with COVID-19 from WES data would be possible, but in clinical practice, this approach requires overcoming several limitations that we also faced in our study. Moreover, WES only analyses exonic variants; therefore, the study may have failed to detect potentially related intronic variants. However, whole-genome sequencing (WGS) is becoming increasingly attractive as an alternative, due to its broader coverage, decreasing cost, and more comprehensive information.

## Methods

**Fig. 3:**
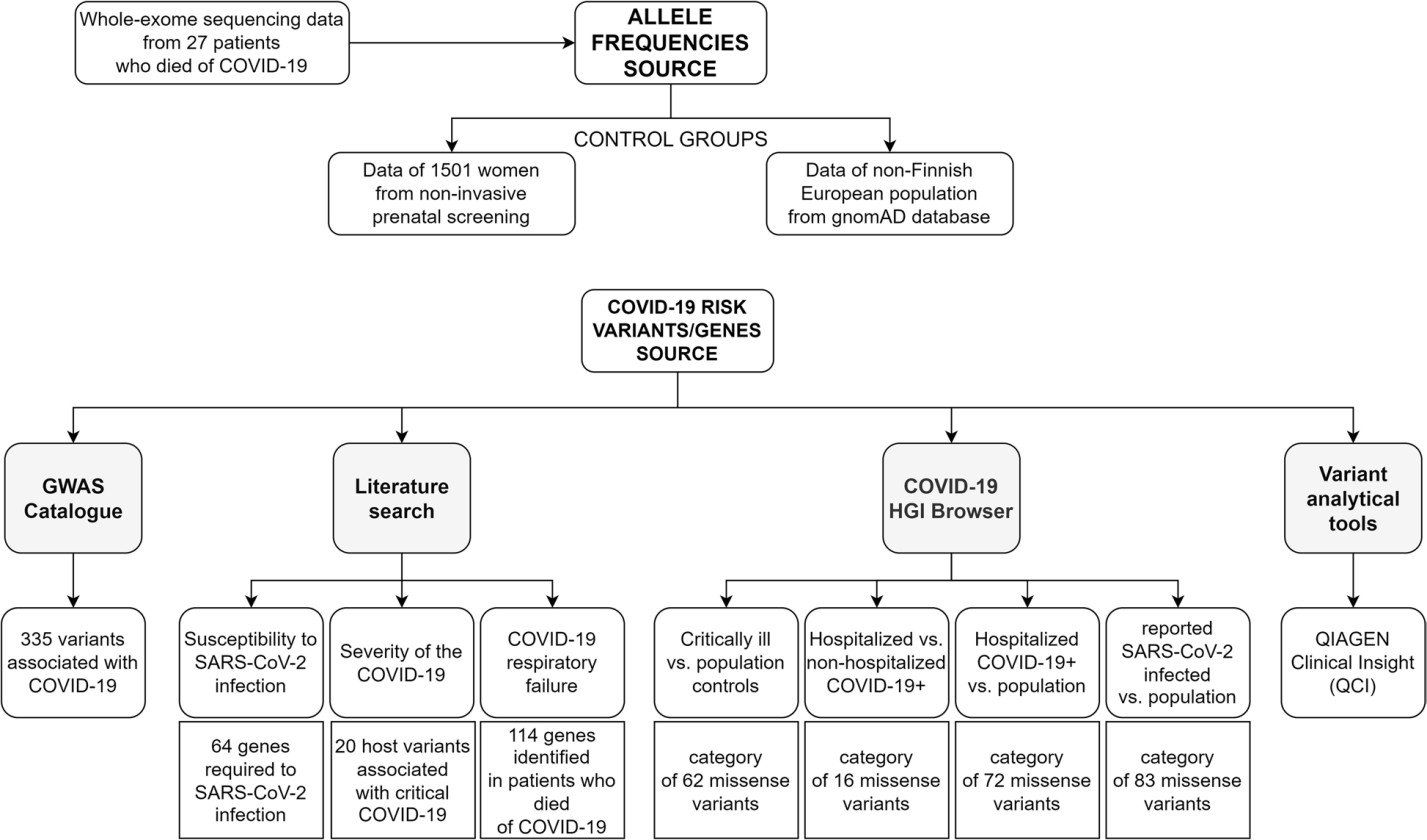
The diagram represents 4 main steps used in identification of risk variants associated with SARS-CoV-2 infections and the severity of COVID-19 disease

The method consists of four main steps, presented in the diagram in Fig. 3. In the first step were analyzed the exome sequencing data derived from pulmonary tissue samples taken at autopsy of 27 (13 men, 14 women) deceased patients, with the mean age 66 years, with a confirmed diagnosis of SARS-CoV-2 infection. The evaluations were performed as part of the autopsy and subsequent laboratory and analytical tasks carried out by the Health Care Surveillance Authority to monitor the course of COVID-19. Performance of the analyses was approved by the relevant ethics committee, N° 21–49, on 8 April 2021. Laboratory processing and bioinformatic analysis of the data were performed as described in Additional file (Methods).

In the second step, we performed a selection of risk variants and/or genes associated with COVID-19. All risk variants associated with COVID-19 found in the GWAS Catalog were downloaded from https://www.ebi.ac.uk/gwas/search?query=COVID-19. We also performed a literature search of risk genes and genetic variants associated with infection and severity of COVID-19 published in 2020 and 2021 via the PubMed database (https://pubmed.ncbi.nlm.nih.gov/). More details are available in supplementary material. COVID-19 HGI GWAS meta-analysis release 6 data was downloaded from https://app.covid19hg.org/. Details for each study are provided on the HGI website and in the consortium paper [43].

In the third step, we compared each group of identified risk variants with our WES data of the deceased patients and with two groups of controls: NIPT data from the Slovak population and data of NFE (v3.1.2, downloaded from https://gnomad.broadinstitute.org/downloads). The detailed information about the NIPT dataset generated and analysed in this study is fully described in our previous studies [45, 55]. Allele frequencies of identified variants for each group (Slovak NIPT data, WES data, and data of NFE) in each analysis were plotted using violin-swarm plots.

In the fourth step, QIAGEN Clinical Insight (QCI™) Analyze software was used to analyse the WES data of the deceased patients. Variants were imported as an industry-standard VCF into the QCI-A web interface, which enables data interpretation. We performed variant filtering based on biological context with “COVID-19” as the keyword which included eight filters.

### Statistical analysis

Python language libraries scikit-allele were used for analysing genetic variation data and matplotlib and seaborn for the graphic representation. The significance of our findings was evaluated using statistical tests implemented in the Python SciPy package. We used the Mann–Whitney U test to compare risk variants allele frequencies from the deceased COVID-19 patients and the Slovak NIPT data or respectively gnomAD data respectively as controls. In addition, observed allele frequencies for each risk variant in all groups were tested using the Fisher's exact test.

## Supplementary Information

**Additional file 1: Table S1. **Genes selected after literature search from 3 types of publications.

**Additional file 2: Table S2. **All 900 Covid-19 associated risk variants used in our analyses with Fisher's exact test p-values. • /p-value - included in the analysis; sig - significance: * - p-value < 0.05, ** - significant after Bonferroni correction; # - overlap with risk variants from QCI infection group; § - overlap with risk variants from QCI critical group; sN - Slovak NIPT data, W - WES data of Slovak dead patients, N - non-Finnish European gnomAD data.

**Additional file 3.** Methods [56–65].

## Data Availability

The NIPT datasets generated and analysed during the current study are available in the DSpace repository, https://dspace.uniba.sk/xmlui/handle/123456789/27. WES datasets are available from the corresponding author on reasonable request.
