# Supplementary material for: Evaluation and limitations of different approaches among COVID-19 fatal cases using whole-exome sequencing data
Source: BMC Genomics. 2023 Jan 10;24:12. doi: 10.1186/s12864-022-09084-5 (PMC9830622; doi:10.1186/s12864-022-09084-5)
Supplement: Supplementary file 3 — Additional file 3. Methods [56–65]. [file 12864_2022_9084_MOESM3_ESM.docx]

## **ADDITIONAL FILE**

**Methods**

## **Whole-exome sequencing analysis**

Genomic DNA from frozen lung tissue samples was isolated using the DNeasy Blood & Tissue Kit (Qiagen, Hilden, Germany) according to the manufacturer's instructions. The concentration of the isolated DNA samples was fluorescently measured with Qubit™ 1X dsDNA High Sensitivity Assay Kits (Life Technologies Corporation, Eugene, Oregon, USA). The integrity of DNA samples was checked by agarose electrophoresis. The genomic DNA samples were sent to the Institute of Applied Biotechnologies laboratory where the Illumina DNA Prep with Enrichment + Twist Comprehensive Exome & Twist mitochondrial DNA panel was prepared from samples. Subsequent sequencing on NovaSeq (Illumina, San Diego, CA, USA) with ≥9,7 Gbp per sample was performed.

We validated the quality of the sequenced reads using reports generated by the tool FastQC 0.11.5 [[56]](https://paperpile.com/c/e9Szc9/O5KX). Next, the reads were aligned to the GRCh38 reference genome with decoy sequences, using the Burrow-Wheeler Alignment tool (BWA-MEM algorithm) 0.7.17  [[57]](https://paperpile.com/c/e9Szc9/ynWt), producing a BAM file for each sample. We sorted the BAM files with SAMtools 1.3.1 [[58]](https://paperpile.com/c/e9Szc9/xIQl) and deduplicated them with Sambamba 0.7.0 [[59]](https://paperpile.com/c/e9Szc9/JWMN), which was also used for indexing the files. The BAM quality was controlled at each step using Qualimap 2.2.2 [[60]](https://paperpile.com/c/e9Szc9/EDbi). Finally, variants were called individually for each sample with the tool DeepVariant 1.1.0 [[61]](https://paperpile.com/c/e9Szc9/9TL8), producing VCF files. Variant analysis was targeted at the regions of the Twist Human Comprehensive Exome Panel defined in the official and publicly available BED file. We assembled, configured and executed all these steps as a variant calling pipeline created in Snakelines 0.11.6 [[62]](https://paperpile.com/c/e9Szc9/oDqx).

In the further analysis we used bcftools 1.8 [[63]](https://paperpile.com/c/e9Szc9/QzPO) to filter the VCF files for passing variants only (their vcf filter value is PASS) and to normalize the files by validating reference alleles and splitting multiallelic records into biallelic variants. Next, the normalized files were merged into a single multisample VCF file which was then annotated with the dbSNP [[64]](https://paperpile.com/c/e9Szc9/fWfl) reference numbers (rs). We also annotated the joint VCF file with the Variant Effect Predictor (VEP) 104.3 [[65]](https://paperpile.com/c/e9Szc9/OgQW).

First, we chose data from a study by Baggen et al. who identified 64 genes that are proviral host factors required to complete the SARS-CoV-2 infection. Subsequently, we filtered out a group of variants located in these 64 genes from our WES data of dead patients. We have identified 253 risk variants in 64 genes from our WES data of dead patients, which were then merged with data from NIPT. Second, we used data from the COVID-19 Host Genetics Initiative [[43]](https://paperpile.com/c/e9Szc9/PFxe) and Pairo-Castineira et al. [[35]](https://paperpile.com/c/e9Szc9/XFVN) which reported genome-wide loci associated with severe manifestations of COVID-19. In these studies we found 20 risk variants, but they were all outside our sample set from WES data of dead patients. Therefore we selected the 15 genes in which these risk variants were found and we identified 277166 risk variants from exome areas (covering a sample set from WES data of dead patients). Third, we used data from Ackermann et al. [[44]](https://paperpile.com/c/e9Szc9/ojV7) which analyzed respiratory failure-associated gene sets. From this study, we selected the GS398329 gene set, which describes genes that are up-regulated in lungs from patients who died from COVID-19. We obtained the list of 114 genes from GeneWeaver (https://www.geneweaver.org/) and identified in them 3076573 risk variants covering exome areas.


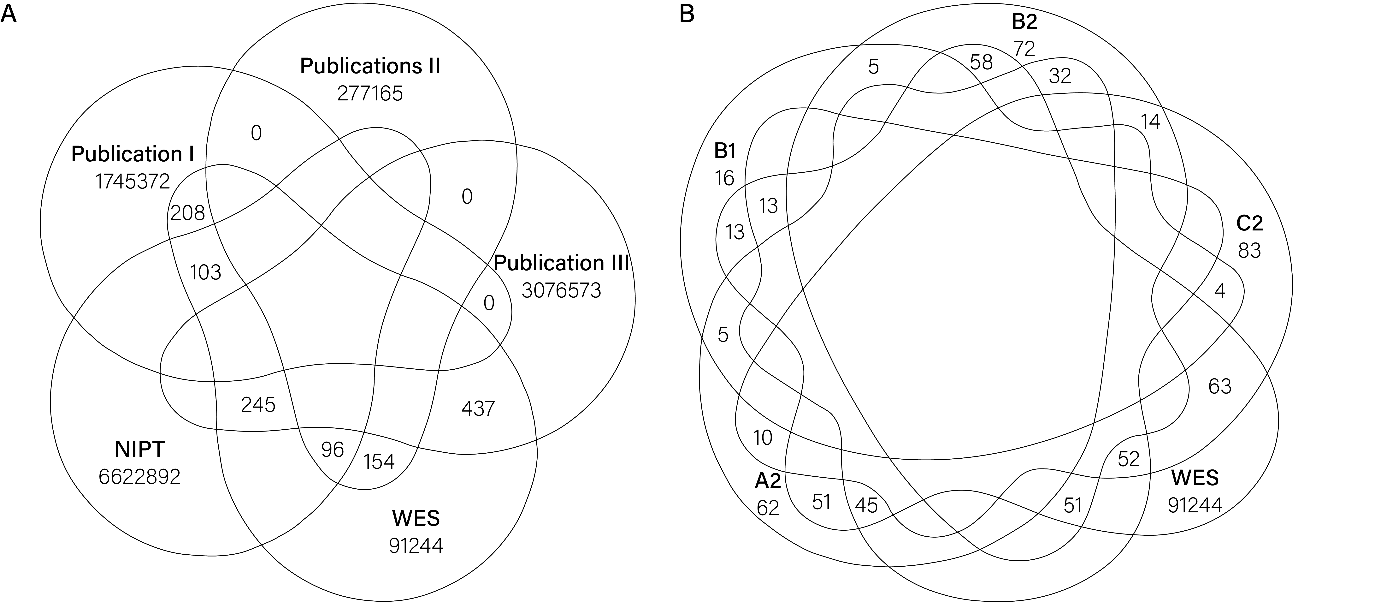


Fig. S1 **A** Venn diagrams for intersection in variants comparison between NIPT, WES data and variants found in publication I. Baggen et al. [[31]](https://paperpile.com/c/e9Szc9/6JV9), II. COVID-19 Host Genetics Initiative [[43]](https://paperpile.com/c/e9Szc9/PFxe) and Pairo-Castineira et al. [[35]](https://paperpile.com/c/e9Szc9/XFVN) and III. Ackermann et al. [[44]](https://paperpile.com/c/e9Szc9/ojV7) **B** Venn diagrams for intersection in variants comparison between NIPT, WES data and missense risk variants identified in individual HGI groups.


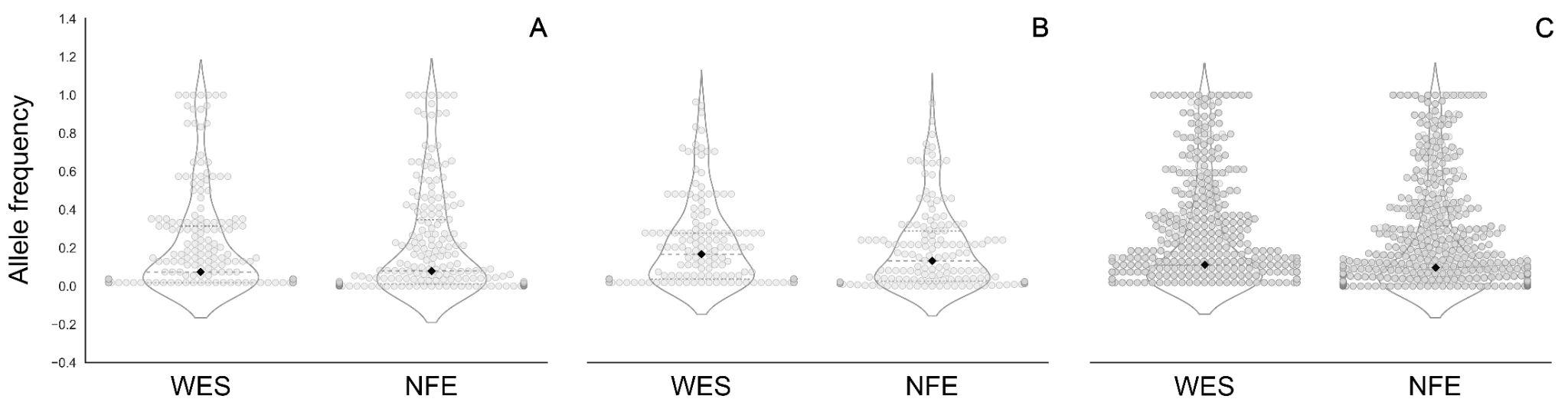


Fig. S2 Violin-swarm plots show allele frequencies for the WES and NFE data identified in 3 types of publications: I. Baggen et al. [[31]](https://paperpile.com/c/e9Szc9/6JV9) (208 risk variants). Statistic: median_W=0.074; st_W=0.067; median_N=0.08, st_N=0.276; Mann–Whitney U test: p-value=0.05776. II. COVID-19 Host Genetics Initiative [[43]](https://paperpile.com/c/e9Szc9/PFxe) and Pairo-Castineira et al. [[35]](https://paperpile.com/c/e9Szc9/XFVN) (154 risk variants). Statistic: median_W=0.167; st_W=0.229; median_N=0.132, st_N=0.214; Mann–Whitney U test: p-value=0.066277. III. Ackermann et al. [[44]](https://paperpile.com/c/e9Szc9/ojV7) (432 risk variants). Statistic: median_W=0.111; st_W=0.278; median_N=0.0.097, st_N=0.279; Mann–Whitney U test: p-value=0.0017173. Median is labeled with ⁭♦; st - standard deviation, W - WES - whole-exome sequencing data from dead patients, N - Non-Finnish European population from the gnomAD database.


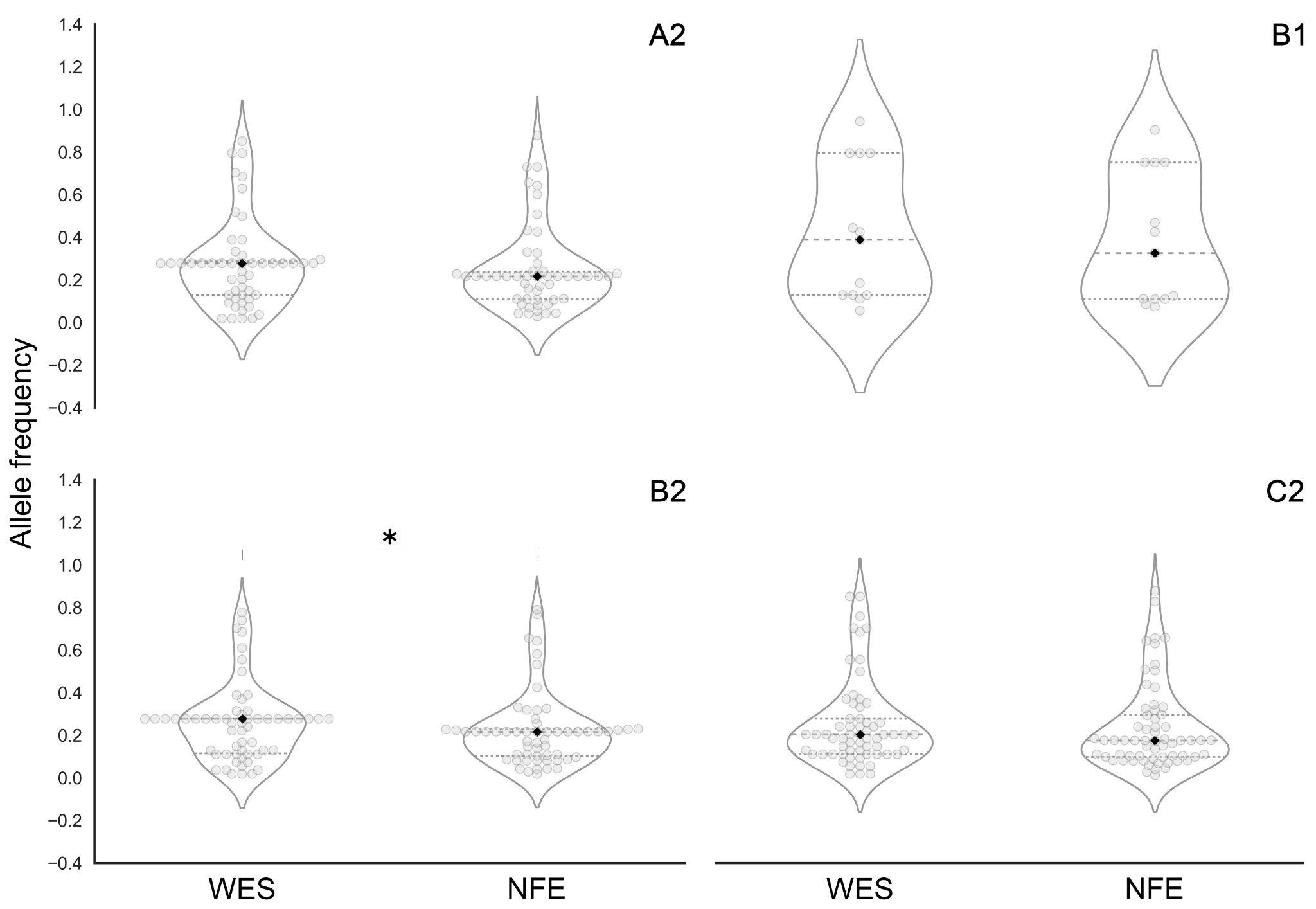


Fig. S3 Violin-swarm plots show allele frequencies for the WES and NFE data. Statistical significance is labeled with *, median with ⁭♦. A2: Critically ill vs. population controls (51 risk variants). Statistics: median_W=0.278; st_W=0.209; median_N=0.217, st_N=0.199; Mann–Whitney U test: p-value=0.08459. B1: Hospitalized COVID-19 vs non-hospitalized COVID-19 (13 risk variants). Statistic: median_W=0.389; st_W=0.321; median_N=0.326, st_N=0.312; Mann–Whitney U test: p-value=0.22057. B2: Hospitalized COVID-19 vs population controls (58 risk variants). Statistic: median_W=0.278; st_W=0.182; median_N=0.217, st_N=0.175; Mann–Whitney U test: p-value=0.03132. C2: Reported SARS-CoV-2 infections vs. population controls (63 risk variants). Statistic: median_W=0.204; st_W=0.203; median_N=0.176, st_N=0.199; Mann–Whitney U test: p-value=0.10934; st - standard deviation, W - WES - whole-exome sequencing data from dead patients, N - Non-Finnish European population from the gnomAD database.
